# Dissecting Motor Neuron Disease With *Drosophila melanogaster*

**DOI:** 10.3389/fnins.2019.00331

**Published:** 2019-04-12

**Authors:** Rachel Walters, John Manion, G. Gregory Neely

**Affiliations:** Dr. John and Anne Chong Lab for Functional Genomics, Charles Perkins Centre, School of Life and Environmental Sciences, The University of Sydney, Sydney, NSW, Australia

**Keywords:** Motor Neuron Disease, *Drosophila melanogaster*, *UAS*/Gal4/Gal80, amyotrophic lateral sclerosis, spinal muscular atrophy

## Abstract

Motor Neuron Disease (MND) typically affects patients during the later stages of life, and thus, MND is having an increasingly devastating impact on diagnosed individuals, their families and society. The umbrella term MND refers to diseases which cause the progressive loss of upper and/or lower motor neurons and a subsequent decrease in motor ability such as amyotrophic lateral sclerosis (ALS) and spinal muscular atrophy (SMA). The study of these diseases is complex and has recently involved the use of genome-wide association studies (GWAS). However, in the case of MND, it has been difficult to identify the complex genetics involved in subtypes, and functional investigation of new candidate disease genes is warranted. *Drosophila* is a powerful model for addressing these complex diseases. The *UAS*/Gal4/Gal80 system allows for the upregulation of *Drosophila* genes, the “knockdown” of genes and the ectopic expression of human genes or mutations in a tissue-specific manner; often resulting in *Drosophila* models which exhibit typical MND disease pathologies. These can then be further interrogated to identify disease-modifying genes or mutations and disease pathways. This review will discuss two common MNDs and the current *Drosophila* models which are being used to research their genetic basis and the different pathologies of MND.

## Introduction

As the global population ages, our societies face an increased prevalence of age-related diseases. A deeper understanding of the biology involved is required to develop new therapies to halt or even reverse disease progression. MND is characterized as the progressive loss of upper and/or lower motor neurons and a decrease in motor ability and function. It has been hypothesized that incorrect synaptic development and function could underlie MND progression (for full review see Murray et al., 2010). Typically, but not exclusively, symptoms begin to develop in the second half of a patient’s life, with incidence peaking between 75 and 79 years of age (Alonso et al., 2009), and leading to a rapid decrease in the quality of life and death (Tabrizi, 2006). The major forms of MND are ALS and SMA.

Genome-wide association studies have been used to establish the heritability and molecular etiology of MND. These studies identify SNPs that show statistical association with a specific phenotype. However, by design GWAS do not typically identify causative mutations (Auton et al., 2015). Instead, these studies flag common SNP variants within haplotype blocks. These regions may contain causative coding mutations, regulatory non-coding mutations, or complex elements that could influence disease susceptibility or progression. Moreover, these associations do not always predict directionality toward disease effect. Significant haplotype blocks are likely enriched for genes that modify disease, or regulatory elements that act in either cis or trans fashion, or even exert effects at long range or via unknown complex mechanisms. To pinpoint any disease-modifying genes within significant disease-associated haplotype blocks, large scale *in vivo* genetic screens are required. *Drosophila* is a well-established model organism through which to perform these screens for the study of MND.

**FIGURE 1:**
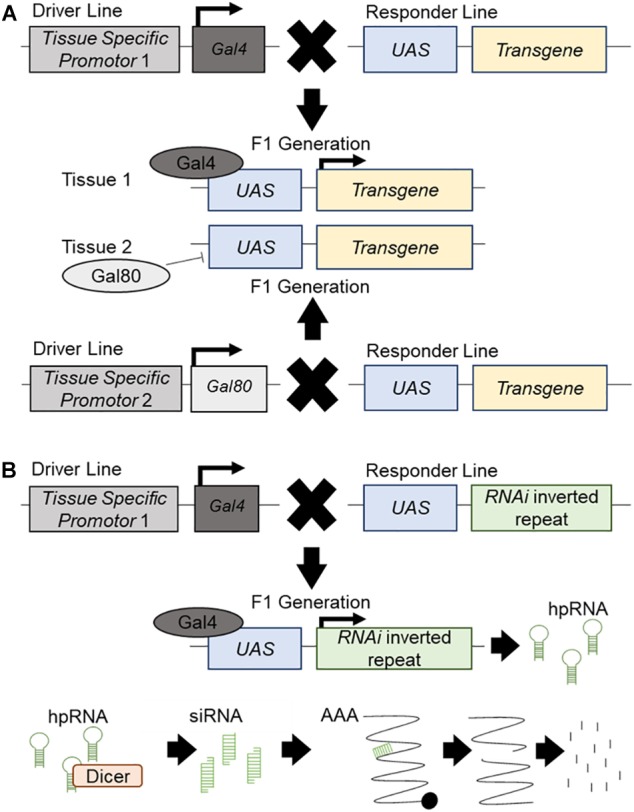
The *UAS*/*Gal4*/*Gal80* system and *RNAi* knockdown. Schematic presentation of the *UAS*/*Gal4*/*Gal80* system and its use in transgene expression and *RNAi* mediated knockdown. **(A)** The driver line contains a different tissue-specific promotor for both *Gal4* and *Gal80*. It will drive the expression of Gal4 or Gal80 in the corresponding tissue. The responder line carries an upstream activator sequence (*UAS*) upstream of a transgene. In the F1 generation, each tissue specific promotor will drive the expression of Gal4 or Gal80 in the respective tissues. Gal80 will bind to the *UAS* and repress its expression in this tissue. Gal4 will bind to the *UAS* and promote the expression of the transgene. **(B)** The driver line contains a tissue-specific promotor which will drive the expression of Gal4 in the specific tissue. The responder line carries a *UAS* upstream of an inverted repeat of an *RNAi* construct. In the F1 generation, the tissue specific promotor will drive the expression of the Gal4 which will bind to the *UAS* and promote the expression of the *RNAi* construct, producing hairpin RNA (hpRNA). hpRNA is processed by Dicer into short interrupting RNA (siRNA) which will attach to endogenous mRNA and create a break in the strand. This broken mRNA will then be targeted for degradation and knockdown of the target gene will occur.

The *Drosophila UAS*/Gal4/Gal80 system is used for tissue-specific targeted regulation of transgene expression (Brand and Perrimon, 1993; Figure 1A), or RNA interference (*RNAi*) inverted repeats designed to “knockdown” genes (Dietzl et al., 2007; Figure 1B). This tool makes *Drosophila* a potent system for the high throughput investigation of candidate disease genes *in vivo*. Many genetic diseases have a root cause of loss-of-function mutations, which is particularly easy to recapitulate using available whole genome *in vivo RNAi* libraries which allow for the targeted knockdown of all corresponding individual genes (Clark and Ding, 2006; Figure 1B). Moreover, disease genes which exert their disease-causing effects via gain-of-function mechanisms may be central to the development and function of the tissue of interest, and thus often will also exhibit loss-of-function phenotypes (Drenth and Waxman, 2007). Establishing an experimental interaction between loss of function phenotypes for candidate disease genes and the underlying disease process can be informative regardless of directionality. *Drosophila* is amenable to rapid and systematic genetic manipulation and is a cost-effective, ethical system to evaluate large gene sets for *in vivo* relevance with respect to organ dysfunction and disease. Another advantage of *Drosophila* models is the high degree of conservation between its genome and that of humans; with around 60% of all genes and 75% of human disease genes having a *Drosophila* ortholog (Adams et al., 2000; Fortini et al., 2000). Furthermore, 76% of human synaptic genes have a *Drosophila* ortholog (Lloyd et al., 2000) and, as it is hypothesized MND is, in part, due to synaptic dysfunction, candidate disease genes and synaptic regulators can be rapidly assessed for function in the well characterized and easily accessible *Drosophila* motor neuron. *Drosophila in vivo* screens allow for the study of morphological changes which occur during MND. The highly accessible *Drosophila* NMJ has been well developed as a model system and this knowledge can be used to study molecular and morphological aspects of synaptic dysfunction (Keshishian et al., 1996). The *Drosophila* glutamatergic NMJ also closely resembles vertebrate glutamatergic central synapses. It has been successfully used to study many aspects of synaptic transmission such as neurotransmitter release (Megighian et al., 2013; Valdez et al., 2015), signaling (Kamimura et al., 2013; Vonhoff and Keshishian, 2017) and homeostatic plasticity (for full review see Frank, 2014). For example, the *Drosophila* NMJ has aided in the discovery of important synaptic genes and proteins such as *Bruchpilot* (Kittel et al., 2006) and Synaptotagmin 4 (Yoshihara et al., 2005) (for full review see Hewitt and Whitworth, 2017). The use of *Drosophila* also allows for an in-depth study of disease progression; from the earliest signs to terminal stages. Finally, the large range of simple assay systems that can be undertaken rapidly using *Drosophila*, from lifespan and motor assays, to anatomical screens, would not be possible in other systems, and can each provide important functional information on the conserved machinery required for proper motor neuron function, and how these systems may be dysregulated during MND.

## Amyotrophic Lateral Sclerosis (ALS)

Amyotrophic lateral sclerosis, the most common form of MND, is a progressive neurodegenerative disease which can be categorized as either sporadic (SALS); cases where no immediate family member is affected, or familial (FALS); cases which have an inherited and often monogenic cause. FALS accounts for just 10% of ALS cases (Kiernan et al., 2011). The worldwide annual incidence is approximately 1.9 per 100,000 (Chiò et al., 2013) with a projected increase of approximately 70% between 2015 and 2040 (Arthur et al., 2016). ALS is ∼1.56 times more common in men than women (Mehta et al., 2014). However, this difference becomes less prevalent with an increase in age, with around the same number of male and female sufferers beyond the age of 70 (Worms, 2001). Symptoms can begin at any stage of life, however, middle age (40–49 years) to elderly (70+ years) are the most common ages at which the disease starts to develop. Following onset, patients suffer a progressive loss of motor function and after diagnosis have an average life expectancy of 5–6 years (Koppers et al., 2012). The pathology of ALS is varied, with patients suffering from symptoms at different time points after diagnostics, this may in part be due to slow prognosis, but, there are three main stages of disease progression; early, middle, and late. Through these stages, the patient will suffer from worsening symptoms and more increased physical weakness (Table 1; The ALS Association of Texas, 2018; Muscular Dystrophy Association, 2019). The leading cause of death in ALS sufferers is respiratory insufficiency. The disease progression of ALS has traditionally been evaluated through phenotypic assessments, however, the phenotypic variability of ALS results in inaccurate measurements and can delay diagnosis. Because of this, the use of multiple biomarkers is now being studied as a novel assessment method for disease progression (for full review see Simon et al., 2014; Huynh et al., 2018).

**Table 1:** The progression of ALS stages, symptoms, and physical effects (The ALS Association of Texas, 2018; Muscular Dystrophy Association, 2019).

Stage	Symptoms	Physical effects
Early	Muscle weakness, fasciculations, and atrophy often limited to one region of the body.	Fatigue, poor balance, slurred words, tripping, and a weak gip.
Middle	The symptoms seen in early stages are more widespread and affect more than one region of the body. Muscles become paralyzed and fasciculations continue.	Muscle contractures, weakness in breathing, and swallowing causing difficulty eating,drinking and breathing.
Late	Most voluntary muscles are paralyzed, and the breathing muscles are very weak.	Very limited mobility, poor respiration causing fatigue, and increased susceptibility to pneumonia. Loss of speech and limited eating/drinking via mouth.


The cause of ALS is thought to be a combination of multiple genetic risk variants and environmental factors including heavy metals (Kamel et al., 2002; Gait et al., 2003) or pesticides (Malek et al., 2012) (for full review see Bozzoni et al., 2016). From twin studies, heritability is estimated to be between 76% (Al-Chalabi et al., 2010)and 61% when known familial cases are excluded (SALS) (Wingo et al., 2011) (for a full review see Al-Chalabi and Visscher, 2014). However, more recent work using genome-wide complex trait analysis (GCTA) has placed the estimate much lower, at between approximately 21% (Keller et al., 2014) and 12% (Fogh et al., 2014). The main reason for the disparity in these estimates is due to the differing methods of obtaining the data, where twin studies often yield higher heritability values compared to GWAS for a variety of complex diseases (Tenesa and Haley, 2013; Feldman and Ramachandran, 2018).

Whilst familial studies have established that mutations in a number of genes can cause ALS, mutations in *C9orf72* (DeJesus-Hernandez et al., 2011), Superoxide Dismutase 1 (*SOD1*) (Rosen et al., 1993), TAR Binding-Protein 43 (*TARDBP*) (Neumann et al., 2006), Ataxin 2 (*ATXN2*) (Elden et al., 2010), and *FUS* (Vance et al., 2009; Table 2) are the most common.

**Table 2:** The major genetic contributors to a patient’s risk of developing ALS.

Gene	Protein	Inheritance	% of FALS cases which exhibit a mutation in this gene	References
*C9orf72*	C9orf72	Autosomal dominant (AD)	23.5	DeJesus-Hernandez et al., 2011
*SOD1*	SOD1	AD	20	Rosen et al., 1993
*TARDBP*	TDP-43	AD	5 (Inclusions in 90% of cases)	Neumann et al., 2006; Mackenzie et al., 2007
*ATXN2*	Ataxin 2	AD	4.7	Elden et al., 2010
*FUS*	RNA-binding protein FUS	AD	5	Vance et al., 2009


To elucidate the contribution of genetic variation to sporadic ALS, population-wide studies have been performed. To date, 14 GWAS have been completed (for full review see Max-Planck-Gesellschaft, 2017) spanning a wide range of populations. These studies have linked many genes to an increased risk of ALS, including, with the exception of *FUS*, those genes discussed above. However, *C9orf72* is the only gene that exhibits a genome-wide significant peak which has been confirmed in a second cohort (Ahmeti et al., 2013). As ALS is heterogeneous in nature, the mechanism by which these or other nearby genes may modify disease is not fully understood and requires further study. These genetic risk factors have well-established *Drosophila* disease models which have routinely been used in the study of ALS, its causes, and its effects on motor neurons.

## C9orf72

**FIGURE 2:**
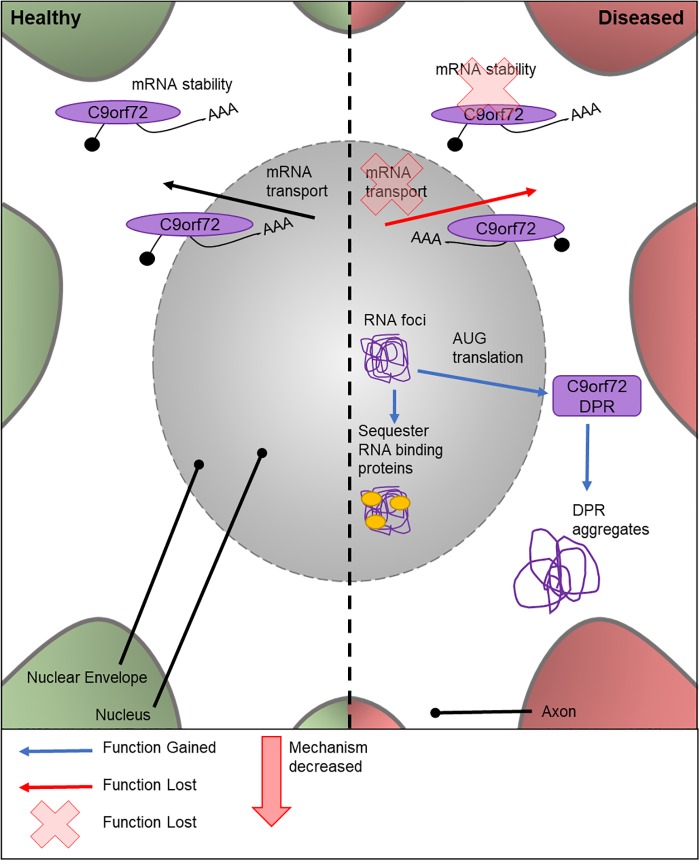
Cellular features of C9orf72 in a healthy and diseased neuron. Schematic presentation of a neuronal cell body with normal and disease-state C9orf72 cellular location and functions. In healthy neurons, C9orf72 is thought to function in mRNA stability and transport. In the diseased state, it is thought to form nuclear RNA foci which sequester RNA binding proteins. Through RAN translation of the mutated gene, DPR proteins are created. These form cytoplasmic protein aggregates. It is also thought mutations could cause the loss of C9orf72’s role in mRNA stability and transport.

The cellular function of C9orf72 is currently under contention. Similar to TDP-43 (discussed below), C9orf72 is thought to play a role in mRNA stability and transport (Figure 2). The first intron of human *C9orf72* contains a hexanucleotide repeat expansion G_4_C_2_. Importantly, an increased number of G_4_C_2_ repeats has been linked to an increased risk of ALS (DeJesus-Hernandez et al., 2011). *C9orf72* genetic mutations are the most frequent known cause of ALS (Majounie et al., 2012); with around 40% of FALS cases exhibiting various numbers of repeat expansions (Renton et al., 2011) and *C9orf72* is also the only locus to show a genome-wide significance in meta-analysis studies (Malek et al., 2012; Al-Chalabi and Visscher, 2014).

There are multiple potential mechanisms by which the G_4_C_2_ repeat expansions can cause disease. These include a loss of C9orf72 normal cellular function, sequestering and altering the function of RNA binding proteins by RNA foci (Lill et al., 2011), or by AUG translated di-peptide repeat proteins (DPRs) (Ash et al., 2013; Figure 2). There is no known *Drosophila C9orf72* ortholog. Therefore, to study the relative importance and contribution of *C9orf72* in MND, transgenic *Drosophila* lines expressing either C9orf72 pure or RNA-only human G_4_C_2_ repeat variants, or animals which express both, have been generated (Mizielinska et al., 2014; Lee et al., 2016). C9orf72 pure lines will express both RNA and DPRs. Ectopic expression of human C9orf72 DPRs in fly motor neurons causes lethality at 25°C, while these models are semi-lethal at 18°C (Yang et al., 2015). The lack of endogenous *Drosophila C9orf72* has proven to be a benefit to these studies, as it provides a control model for experimental use which contains no C9orf72.

Studies suggest that the C9orf72 RNA species form nuclear RNA foci, which could have a role in sequestering RNA-binding proteins in the nucleus (Ash et al., 2013). Furthermore, the G_4_C_2_ repeat expansions, but not the RNA foci (Mizielinska et al., 2014), are translated via repeat-association non-ATG translation to form DPR proteins, which form aggregates in the cytoplasm of the cell body (Mackenzie et al., 2013; Mori et al., 2013; Zu et al., 2013; Cooper-Knock et al., 2014). A further model has been developed, using a *UAS*-(G_4_C_2_)_48_ construct, to study the effect of G_4_C_2_ repeats on translation (Burguete et al., 2015). This highlighted that these repeats localize in neurites and can negatively impact the branching of the cell, ultimately affecting the neuron’s function. Mizielinska et al.’s (2014) study also suggests the pathogenicity of C9orf72 DPRs is highly associated with arginine containing DPR proteins, adding to the growing body of knowledge on DPR-specific pathology. The effects of RNA foci in the neuron has also been investigated (Burguete et al., 2015). However, the link between RNA and disease progression is widely debated. *Drosophila* lines expressing only the RNA foci have recently been created (Moens et al., 2018) for the study into the role of repeat RNA on disease progression without the input from the DPR proteins. Depending on the genomic location of the RNA repeats, lines can be produced with cytoplasmic or nuclear foci, allowing the elucidation of these different pathologies. These early RNA-only models have suggested that neither cytoplasmic or nuclear RNA are toxic and therefore has a limited role in ALS (Moens et al., 2018). *Drosophila* models have also shown that changes to transport through the nuclear pore, via the nuclear-pore complex, contribute to neurodegeneration in C9orf72 pathogenesis. This was validated *in vitro* using patient neurons. However, the cause of the defective transport has yet to be found. Zhang et al. (2015) attributes it to sense RNAs, whereas Freibaum et al. (2015) suggests it could be due to a combination of DPRs and toxic RNAs. As shown here, there have been many attempts to elucidate the contribution of different possible mechanisms responsible for C9orf72-caused disease progression. These studies highlight the ongoing effort to establish a timeline and mechanism for C9orf72 toxicity using genetically tractable systems.

## SOD1

**FIGURE 3:**
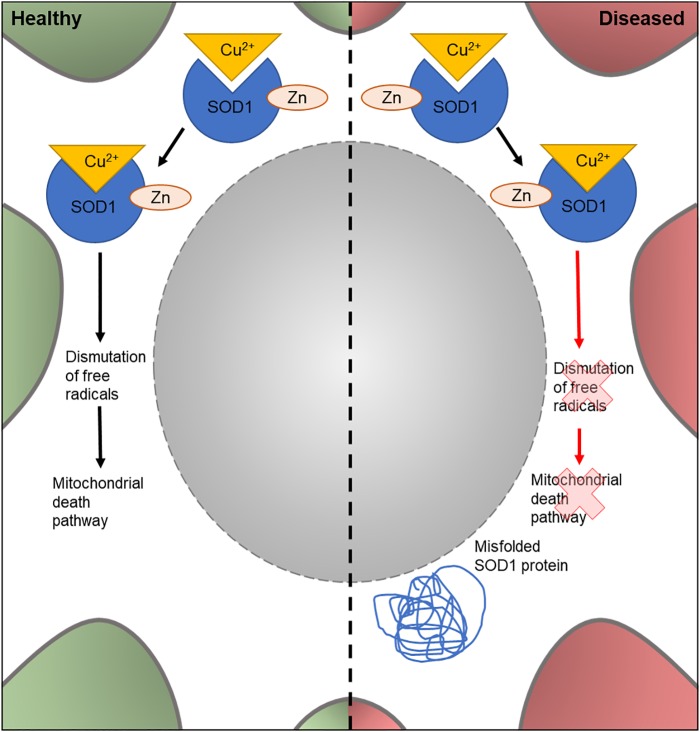
Cellular features of SOD1 in a healthy and diseased neuron. Schematic presentation of a neuronal cell body with normal and disease-state SOD1 cellular location and function. In healthy neurons, SOD1 is located in the cytoplasm. Here, it binds to Cu^2+^ and Zn to form a complex involved in dismutation of free radicals; part of the mitochondrial death pathway. In diseased neurons, mutated SOD1 is also found in the cytoplasm. However, it loses its dismutation function and has an unknown gain of function. It can also form misfolded protein aggregates in the cytoplasm.

The human *SOD1* gene encodes one of three members of the SOD enzyme family. As shown in Figure 3, SOD1 proteins bind to Cu^2+^ ions via a specific binding site and catalyze the dismutation of free radical species in the cell. SOD1-mediated removal of harmful superoxides is hypothesized to suppress apoptosis and prevent cellular damage by free radicals (Danial and Korsmeyer, 2004; Bunton-Stasyshyn et al., 2015). It was the first gene to be linked to familial ALS, with 11 missense *SOD1* mutations showing an association with ALS (Redler and Dokholyan, 2012). Currently, over 90 disease-modifying mutations have been found in *SOD1* (Redler and Dokholyan, 2012; Bunton-Stasyshyn et al., 2015) and around 20% of FALS cases carry a *SOD1* mutation (Shaw et al., 1997). Although the mechanism(s) through which the mutated enzyme contributes to ALS are unknown, there is evidence that the accumulation of misfolded proteins and a gain of toxic function of the mutated enzyme is involved (Clement et al., 2003; Beers et al., 2006; Nagai et al., 2007).

A recent *Drosophila SOD1* model (Gallart-Palau et al., 2016) was found to exhibit non-functioning mitochondria, a cellular phenotype of ALS. In this model, the human wildtype (*hSOD*) and mutant *SOD1*^G93A^ was expressed in the thoracic muscles of *Drosophila*. This targeted expression lead to phenotypes which are stereotypical of ALS, such as impairment of normal motor behavior and mitochondrial pathology. This study builds on previous work, where transgenic motor neuron specific expression of *hSOD1* exhibited progressive negative motor effects accompanied with defective neural circuit electrophysiology, accumulation of SOD1 aggregates and an increased stress response in ventral nerve cord glial cells (Watson et al., 2008). These studies suggest that overexpression of mutated SOD1 protein can cause both cell autonomous and non-cell-autonomous damage, as in the case of the effect on glial cells (Watson et al., 2008), and is a possible cause of *SOD1*-mediated ALS. However, in these studies, it is not clear whether the disease-phenotypes observed are due to overexpression of the mutant *SOD1*, or from the mutated protein itself and therefore would be caused by low-levels of the mutated protein. To address this issue, a recent study has investigated the effect of dosage of transgenes on subsequent disease pathology in *Drosophila* models of *SOD1*-associated ALS (Şahin et al., 2017). Through the mutation of endogenous *Drosophila SOD1* and comparing with multiple copy number insertions, the study demonstrated a predominance of gain of function mutations. Moreover, this new model allows the study of *SOD1* in ALS pathology at typical expression levels by utilizing the endogenous genetic machinery. Together this highlights the diversity of *Drosophila* disease models and their importance in investigating disease-linked mutations and their effect at the protein level.

## TDP-43

**FIGURE 4:**
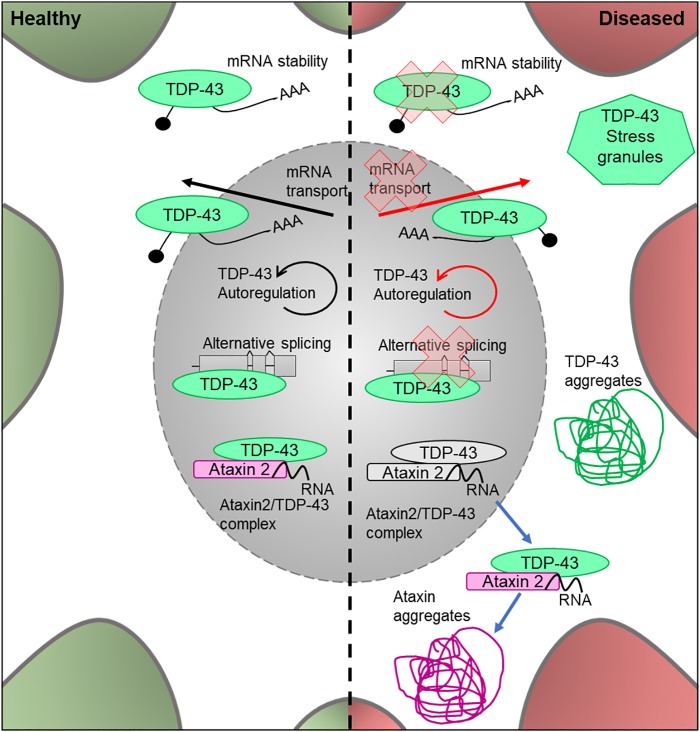
Cellular features of TDP-43 and Ataxin 2 in a healthy and diseased neuron. Schematic presentation of a neuronal cell body with normal and disease-state TDP-43 and Ataxin 2 cellular location and functions. In healthy neurons, TDP-43 is located in both the cytoplasm and the nucleus, where it undergoes autoregulation. It is an RNA-binding protein which is involved in alternative splicing, mRNA transport and cytoplasmic stability of the mRNA. In healthy neurons, Ataxin 2 is located in the nucleus and modifies TDP-43 activity by forming a complex in the presence of RNA. In the disease state, the protein loses its ability to autoregulate as well as its function in alternative splicing, mRNA transport and mRNA stability. It forms ubiqitinated TDP-43 aggregates in the cytoplasm and is known to associate in stress granules. In the disease state, the Ataxin 2/TDP-43 complex translocates to the cytoplasm where it forms TDP-43 and Ataxin 2 aggregates.

TDP-43 (encoded by *TARDBP*) is a DNA- and RNA-binding protein which acts as a nuclear transcription factor and is thought to bind a large proportion of the transcriptome. It has been implicated in the transport of mRNAs to dendritic granules (Wang et al., 2007) and RNA metabolic processes (Buratti and Baralle, 2010; Figure 4). Homozygous *TDP-43* knock-out causes peri-implantation lethality in mouse embryos (Wu et al., 2009), and utilizing the Cre/Lox system to induce TDP-43 knockout later in life is also lethal (Chiang et al., 2010). The Cre/Lox system allows for time- and/or tissue-specific activation or repression of target genes in mouse models and can therefore be used to study mutations in adult animals and their developed organs and tissues. In this system, tamoxifen acts as an inducer at a mutated estrogen receptor (Cre/ERT2), triggering recombination and deletion (Feil et al., 2009). This allows for temporal-specific inactivation of target genes. This demonstrates that TDP-43 is essential for development, as well as throughout adult life. In 2006, TDP-43 was found to be the characteristic ubiquitinated protein found in neuronal cytoplasmic inclusions, a stereotypical neuropathological feature of ALS (Neumann et al., 2006; Figure 4). TDP-43 pathology likely stems from the translocation of this protein from the nucleus into the cytoplasm, where it forms ubiquitinated aggregates (Arai et al., 2006). This has led to two possible pathogenic mechanisms being explored; a gain-of-function of the protein once translocated to the cytoplasm, possibly due to fragmentation and phosphorylation (Arai et al., 2006), and a loss-of-function mechanism in the nucleus due to mutation of the gene (Giordana et al., 2010). However, there is additional evidence that supports a combination of these pathways to be responsible for the proteinopathy (Lee et al., 2012).

*TARDBP* is highly conserved between *Drosophila* and humans, and the fly homolog is called *TBPH* (Lukacsovich et al., 1999). This similarity allows for in-depth study of the role *TDP-43* mutations play in ALS etiology. Both loss and gain of function *TDP-43* models have been shown to negatively affect lifespan, motor function and synaptic transmission in *Drosophila* (Feiguin et al., 2009), all of which are common characteristics of ALS pathology (Diaper et al., 2013) showing that *Drosophila* can be successfully used to model this disease-associated gene. Moreover, these models have shown that manipulation of TDP-43 levels by either loss (mutant) or gain of function (overexpression of wildtype *TBPH*) leads to toxicity. A recent loss-of-function (*TBPH* null mutant) fly larval model suggests that *TBPH* binds to the pre-mRNA form of the gene and regulates its levels by preventing degradation of the transcripts (Chang et al., 2014). *Drosophila* models have been successfully utilized to suggest a mechanism by which *TBPH* proteins form aggregates in the cytoplasm of the cell. Other disease mechanisms have been discovered through the use of *Drosophila* disease models, for example, that synaptic transmission is an early event in the onset of ALS (Diaper et al., 2013). Both loss- and gain-of-function models have been shown to have impaired synaptic transmission in larval and adult models at the pre-synaptic bouton and a progressive loss of function phenotype (Chang et al., 2014). Moreover, a loss of *TBPH* can also lead to reduced cacophony protein levels. *Cacophony* encodes the alpha 1 subunit of the presynaptic calcium channel responsible for the presynaptic voltage gated Cav2 current. It is localized at the active zones of the NMJ and other synapses (Kawasaki et al., 2004; Peng and Wu, 2007) and is required for neurotransmitter release at the NMJ and for synaptic growth (Rieckhof et al., 2003). Due to its importance in NMJ function, *cacophony* dysregulation following TDP-43 loss may be an important disease mechanism in ALS and warrants further investigation. The discoveries highlighted here show the power of using *in vivo* fly models to study TDP-43 and its role in MND.

## Ataxin 2

**FIGURE 5:**
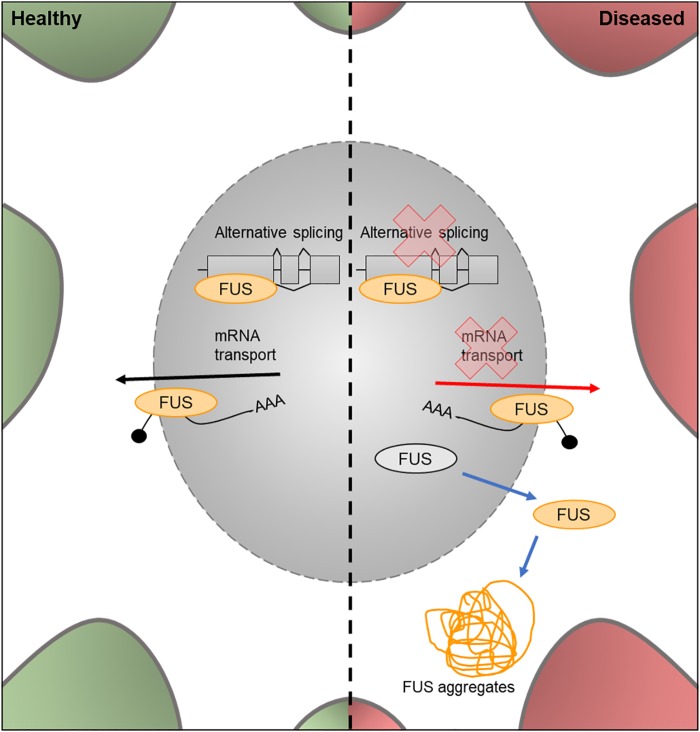
Cellular features of FUS in a healthy and diseased neuron. Schematic presentation of a neuronal cell body with normal and disease-state FUS cellular location and functions. In healthy neurons, FUS is located in both the nucleus and the cytoplasm and is involved in alternative splicing of pre-mRNA and mRNA transport out of the nucleus. In the disease state, FUS translocates into the cytoplasm and loses its function in splicing and mRNA transport. It can also form FUS aggregates due to phase transitioning.

Ataxin 2 is thought to act as a modifier of TDP-43 toxicity, binding to TDP-43 in the presence of RNA and forming a nuclear complex of unknown function. In ALS sufferers, the complex is translocated into the cytoplasm where it contributes to the formation of TDP-43 protein aggregates (Elden et al., 2010; Figure 5). Thus, it is important to understand the interaction between these proteins, and whether mutations in *ATXN2* play a causal role in TDP-43 toxicity in ALS.

Mutations which cause intermediate-length polyQ expansions (30Q) in *ATXN2* are significantly associated with ALS. It has been shown that the upregulation of the *Drosophila* homolog of *ATXN2* increased the toxicity of TDP-43 via increased accumulation of TDP-43 aggregates (Elden et al., 2010; Figure 5). These effects were observed in retinal structures and lead to decreased mobility and an overall reduction in lifespan. These results showed that the ability of *ATXN2* to modulate TDP-43 toxicity is conserved through to *Drosophila*, again showing the value of this system to help understand the molecular events leading to MND.

However, *TDP-43* is not the only ALS-associated gene which *ATXN2* has been found to interact with. When *ATXN2* (30Q) intermediate expansions are co-expressed with a depletion in C9orf72, there is an increase in *ATXN2* aggregates and subsequent cell death (Ciura et al., 2016). Further, evaluation of *ATXN2* as a disease modifier in patients carrying a *C9orf72* expansion mutation suggests that patients with both intermediate *ATXN2* repeat lengths and *C9orf72* expansions are more susceptible to ALS development (van Blitterswijk et al., 2014). Together these studies support a complex mechanism for the onset and development of ALS, involving many genes and their interactions. With the power of the fruit fly genetic toolbox, these interactions can be rapidly characterized *in vivo*.

## FUS

**FIGURE 6:**
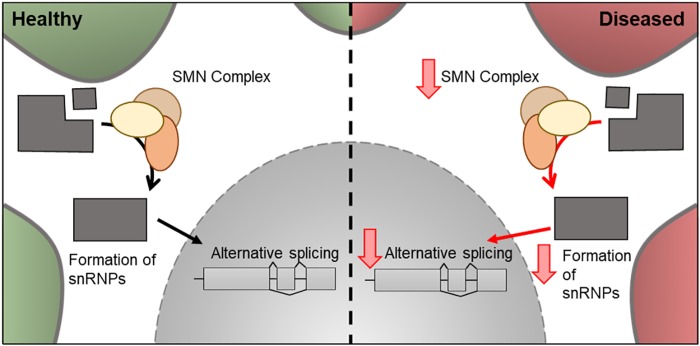
Cellular features of *SMN1* in a healthy and diseased neuron. Schematic presentation of a neuronal cell body with normal and disease-state SMN cellular location and functions. In healthy neurons, SMN is located in the cytoplasm where it forms a complex involved in the assembly of snRNPs. These are essential for pre-mRNA splicing in the nucleus. In the disease state, the levels of SMN decreases and therefore there is a decrease in its downstream cellular functions.

The *FUS* gene encodes an RNA binding protein, a component of the hnRNP complex. This protein is involved in the splicing of pre-mRNA and the export of processed mRNA into the cytoplasm from the nucleus (Vance et al., 2009; Figure 6). Mutations in the nuclear import factor signal of the *FUS* gene results in neuronal cytoplasmic mislocalization of FUS protein (Darovic et al., 2015) and these mutations have been observed in approximately 3% of FALS cases (Kwiatkowski et al., 2009).

The *Drosophila* ortholog of *FUS* is *Caz* and the mutants *Caz* P398L and Q349X are currently used as disease models for *FUS*-related ALS research. For example, expression of fly *Caz*, human wild-type or disease-relevant variants of *FUS* promote cytotoxicity (Jäckel et al., 2015). To study the function of *Caz* at the NMJ Machamer et al. (2014) used overexpression of wildtype *Caz* and mutant human *FUS*. It was suggested that *Caz*/*FUS* modulates the structure and function of the NMJ. Additionally, the overexpression of human *FUS* resulted in a decrease in presynaptic active zones and impaired synaptic transmission; possibly through a dominant-negative mechanism or by the downregulation of endogenous *Caz*. However, this finding was not repeated in wildtype *Caz* experiments, highlighting that overexpression of human genes in the fly may cause toxicity which is independent of disease-associated mutations. This finding was supported by another study, which showed disease-independent toxicity due to overexpression of human *FUS* (Xia et al., 2012). *Drosophila* has also been used to assess the role of *FUS* in axonal transport (Baldwin et al., 2016), which was affected in both *FUS* and *Caz* loss and gain-of-function models through perturbed vesicle and mitochondrial transport. It has also been shown that *Caz* mutants have severe developmental and locomotor defects which, in overexpression models, became more prominent with age, a stereotypical ALS phenotype. As both the loss and gain-of-function models affected the structure and function of the axon, it can be suggested that *Caz* is critical, and either loss or gain may result in a neurodegenerative phenotype.

Recent work has highlighted the mechanism of phase-transition in RNA-binding proteins and it is believed to play a role in RNA-binding protein-linked neurodegeneration (for full review see Harrison and Shorter, 2017). A *Drosophila* model of FUS-linked ALS has shown that this RNA-binding protein undergoes phase-transitioning into solid aggregates due to its prion-like binding domain and interactions with arginine-rich domains (Bogaert et al., 2018) and the effect of this on MND phenotypes and possible reversal of the transition has been further studied. Guo et al. (2018) have shown that in ALS-linked *FUS*-mutation overexpression models there is a higher level of neurodegeneration and a decrease in lifespan. However, these disease phenotypes can be rescued by overexpression of Kapβ2, a nuclear-import factor which reverses the production of FUS aggregates via phase-transitioning through prevention and disassociation of the formed aggregates.

## Spinal Muscular Atrophy (SMA)

Spinal muscular atrophy is one of the leading genetic causes of infant mortality in the United States (Miniño et al., 2010) and results in the degeneration of the anterior motor neurons, progressive atrophy of the muscles and eventually respiratory failure and death (Lefebvre et al., 1995). It has an estimated incidence of between 1 in 6000 and 1 in 10,000 live births (Ogino et al., 2002) and a carrier frequency of 1/40 to 1/60 (Prior et al., 2010). There are five main forms of SMA, SMA0 (Pre-natal SMA), SMA1 (Werdnig-Hoffmann disease), SMA2 (Dubowitz disease), SMA3 (Kugelberg-Welander disease), and SMA4. These sub-types manifest themselves at different stages of life; prenatal, 0–6 months, 6–18 months, over 12 months and in adulthood, respectively (Pearn, 1980). The most severe form of SMA is SMA0 where death usually occurs rapidly after birth (Grotto et al., 2016). SMA1 is the next most severe, where patients cannot sit upright unaided and often do not survive past their second year. SMA2 and SMA3 are less severe and SMA4 is the least severe of the subgroups, with patients being able to walk unaided and only having mild symptoms (Munsat and Davies, 1992). Although they have different severities and ages of onset, the subgroup of an SMA sufferer is not considered when selecting patients for clinical trials as often endpoint and interventions-used are a more important factor. To date, the only drug which has been approved to treat SMA in the United States is Spinraza^TM^ (Nusinersen). In clinical trials, administering Spinraza^TM^ every 4 months resulted in an improvement in motor function in ∼60% of infants (FDA, 2016).

In 1995, mutations in exon 7 of the *SMN1* gene and resulting decrease in SMN protein levels was associated with SMA (Lefebvre et al., 1995). Around 95% of SMA cases are caused by the homozygous absence of *SMN1* (Sugarman et al., 2012). *SMN1* is located near the telomere of chromosome 5, whereas *SMN2*, a nearly identical copy of *SMN1* is located closer to the centromere and, due to a silent C-to-T transition (C840T), undergoes alternative splicing to create a truncated protein, which is rapidly degraded in the cell (Burnett et al., 2009). Although both *SMN1* and SMN2 produce SMN protein, only 10% of *SMN2* transcripts produce functional protein (Monani et al., 1999). A decrease of SMN protein is directly correlated with the classification of SMA and severity of the disease, with reductions in levels of ∼70, ∼50, and ∼30% in types 1, 2, and 3, respectively (Crawford et al., 2012). The copy number of the *SMN2* gene, also increases with sub-group classification with SMN0 patients having only one copy of the gene (Butchbach, 2016). This suggests *SMN2* can play a disease-modifying role in the severity of SMA (Crawford et al., 2012). The mechanism of actions of Spinraza^TM^ is to increase the levels of SMA and it contains an anti-sense oligonucleotide directed to *SMN2*, used to increase the levels of functional SMN protein produced in the cell (Zanetta et al., 2014). SMN is a cytoplasmic protein involved in the assembly of snRNP, which are essential for pre-mRNA splicing (Figure 6). It has been hypothesized that the mechanism which leads to SMA is either a loss-of-function of SMN-mediated snRNP assembly role, resulting in alternative splicing of target genes (Gabanella et al., 2007) or a loss-of-function of mRNA transport in neurons (Fan and Simard, 2002; Eggert et al., 2006). However, other functions related to the actin-cytoskeleton, such as neurite outgrowth, NMJ formation, and profilin binding, have also been implicated in SMA pathology (Giesemann et al., 1999; Chan et al., 2003; Rossoll et al., 2003).

The *Drosophila* genome has one ortholog of *SMN1* (*DmSMN*) (Miguel-Aliaga et al., 2000; Chan et al., 2003). *Drosophila* models utilizing a mutated *DmSMN*^73Ao^ have been used to show that reduced levels and activity of SMN protein causes the inability to fly or jump, morphological defects at the NMJ, and lethality (Chan et al., 2003; Chang et al., 2008). This shows that SMA pathology can be successfully modeled in *Drosophila*. Null mutants and *RNAi* lines have been used to reduce SMN levels by different degrees, mimicking the levels of SMN found in different SMA classifications and recapitulating the disease more thoroughly. These models have been successfully used to further classify the role of the SMN protein (Chan et al., 2003) and its specific binding capability to snRNPs (Garcia et al., 2016). Recent work suggests an additional role for SMN in regulating the actin cytoskeleton (Braun et al., 1995; Rajendra et al., 2007). By showing that *DmSMN* mutants require protein rescue in both nerves and muscle tissue to save normal motor function, these studies suggest a muscle-specific role and a novel disease-causing pathway in the muscle tissue. SMN may act as a sarcomeric protein which is required for the expression of muscle-specific actin, its organization and the subsequent formation of muscle tissue, something which is lacking in SMA patients. However, it has also been suggested that muscle degeneration can also occur due to lack of innervation (Ling et al., 2012), highlighting the important role SMN has in the nervous system. Further research is required to discern whether incorrect muscle formation is due to a motor neuron-specific role of SMN and a subsequent lack of innervation, or whether a muscle-specific novel function of SMN also contributes to SMA pathology. This highlights the importance of the overall view of SMA pathology to establish a timeline of the disease and the role of correct muscle cell function in the severity of SMA. The use of *Drosophila* in the study of SMA will allow for future study into tissue-specific rescue models and the analysis of the timeline of SMA pathology to elucidate the pathology and molecular etiology of this disease.

## Conclusion

With the completion of the *Drosophila* genome sequence in 2000 (Adams et al., 2000), and the publishing of the first draft of the human genome in 2001 (International Human Genome Sequencing Consortium [IHGS], 2001; Venter et al., 2001) the high level of conservation of genes between fruit flies and humans was established, and the benefit of systematic genetic dissection of molecular pathways in the fruit fly was reinforced. The use of *Drosophila* genetics has a long and successful history in the study of various human genetic diseases, ranging from cancer biology (Simon et al., 1991; Olivier et al., 1993) to cardiovascular disease (Kim et al., 2010; Neely et al., 2010), from Parkinson’s (Feany and Bender, 2000; Song et al., 2017) to Alzheimer’s (Kilian et al., 2017). The studies discussed here highlight the successful research efforts into understanding MND pathologies but also show possible future directions of research efforts; which are needed to increase our understanding of MND and to find novel therapeutics. As a model organism, *Drosophila* provides a fast, cheap, and powerful platform to define the genetic underpinnings of complex human diseases and should be continually used in the investigation into MND.

## Author Contributions

All authors contributed to the writing of the manuscript and read and approved the final manuscript.

## Conflict of Interest Statement

The authors declare that the research was conducted in the absence of any commercial or financial relationships that could be construed as a potential conflict of interest.

## Abbreviations

ALS: amyotrophic lateral sclerosis
*C9orf72*: chromosome 9 open reading frame 72
*Caz*: *Cabeza*
DPR: di-peptide repeat(s)
FALS: familial amyotrophic lateral sclerosis
*FUS*: fused-in-sarcoma
G_4_C_2_: GGGGCC
GWAS: genome-wide association study(ies)
hnRNP: heterogeneous nuclear ribonucleoprotein(s)
*hSOD*: human SOD
MND: Motor Neuron Disease(s)
NMJ: neuromuscular junction
snRNP: small nuclear ribonucleoprotein(s)
SALS: sporadic amyotrophic lateral sclerosis
SMA: spinal muscular atrophy
*SMN1*: survival of motor neuron 1
SNP: single nucleotide polymorphism(s)
SOD: superoxide dismutase
*TBPH*: TAR DNA binding homolog
